# Supplementary figures and images for: The impact of model assumptions in interpreting cell kinetic studies
Source: PLoS Comput Biol. 2025 Jun 3;21(6):e1012704. doi: 10.1371/journal.pcbi.1012704 (PMC12133179; doi:10.1371/journal.pcbi.1012704)

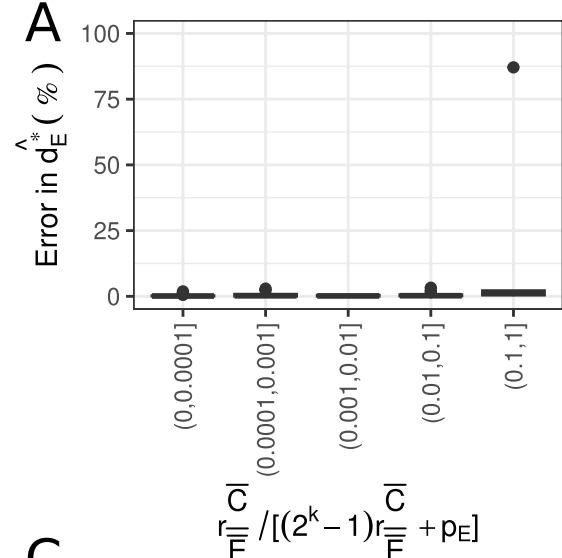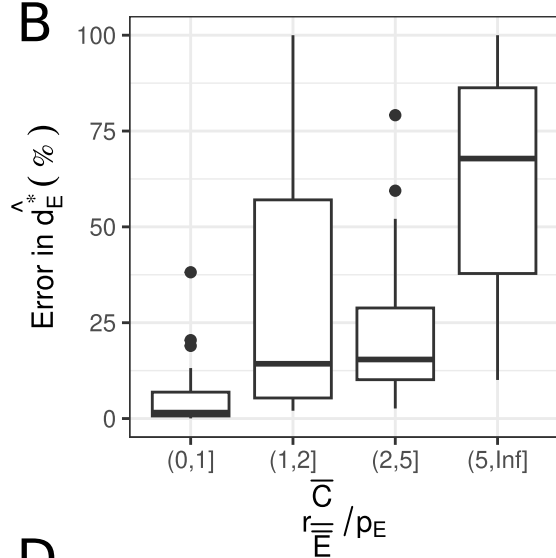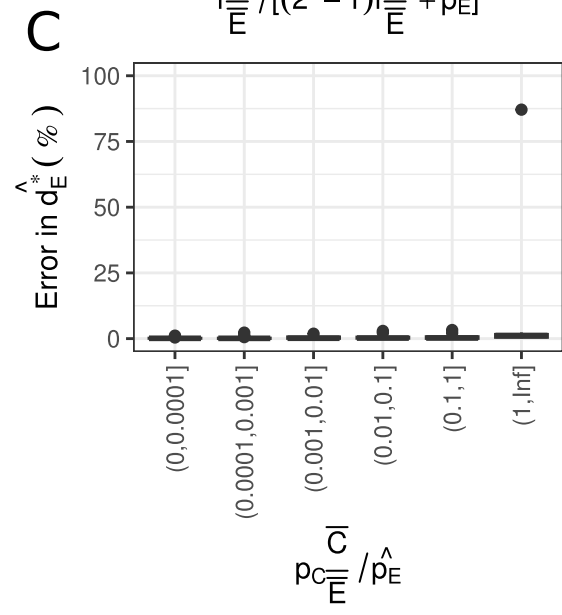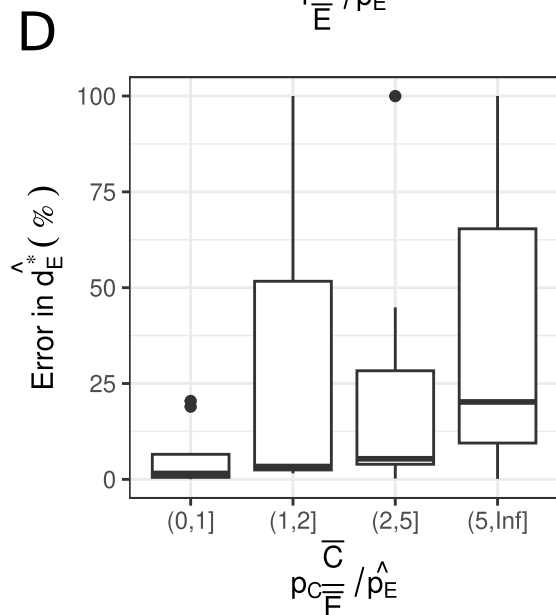

Supplement: S1 Fig — (PDF) [file pcbi.1012704.s001.pdf]

A

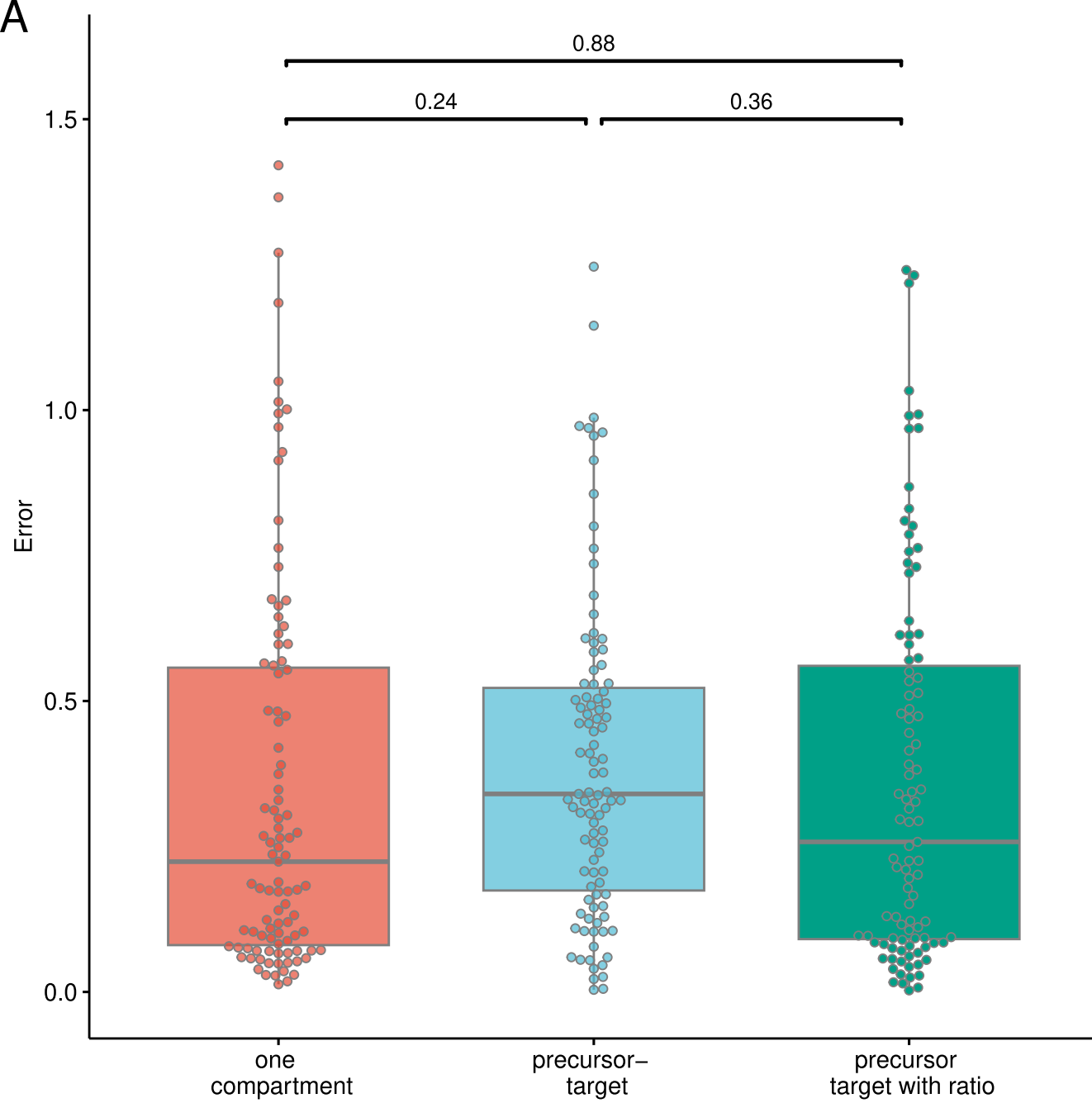

B

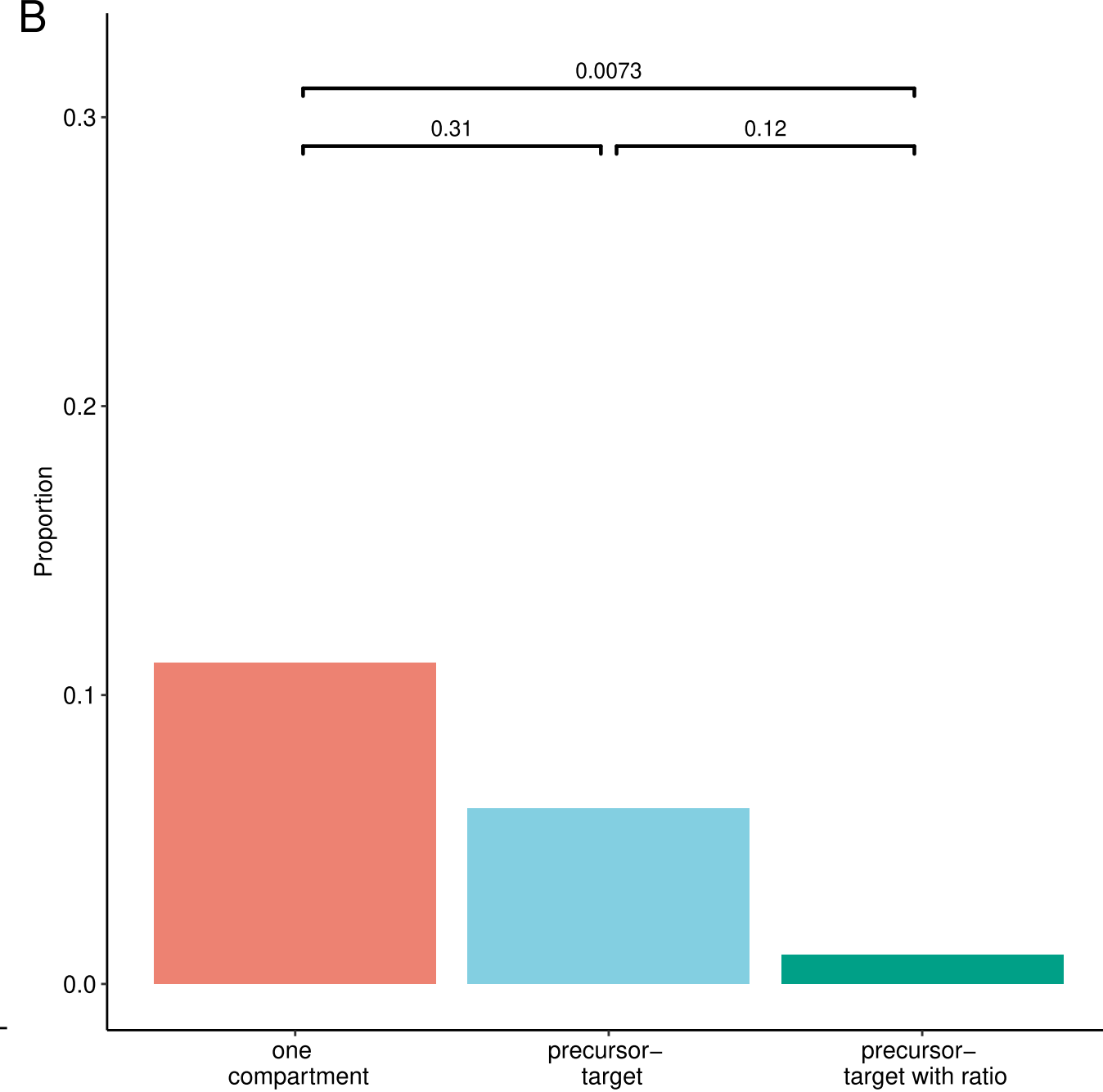

Supplement: S3 Fig — This shows the same data as in Fig 6A but without the y axis truncation. (PDF) [file pcbi.1012704.s003.pdf]

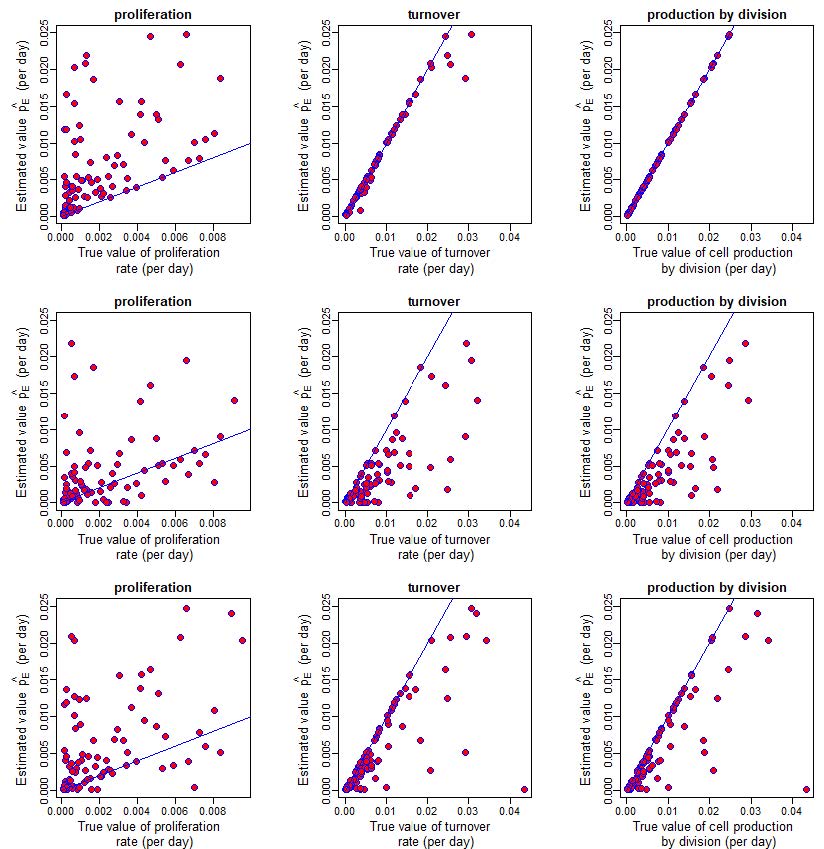

Supplement: S4 Fig — Discrepancy between point estimate of the proliferation rate and the true value (expressed as a fraction of the true value) for the one compartment model (red), precursor/target model (blue) and precursor/target with ratio model (green).On increasing the values of pE used to generate the labelling data two effects were noticed compared to the results depicted in Fig 6: first, errors were now much lower, second, there was no longer any significant difference in the size of the errors associated with each of the three models. P values are calculated by Wilcoxon signed rank, two-tailed; not corrected for multiple comparisons (number of independent comparisons ≤ 3). B 95% confidence intervals (CI) were estimated by bootstrapping the data (Methods) and the fraction of runs where the true value lay within the CI was reported. Colours as for A. Compared to the corresponding figure for realistic data but with a lower value of pE (Fig 6) the proportion of runs where the estimate fell within the CI was still very low but the pattern across the models was quite distinct, for this set of parameters the one compartment model outperformed the other two models. (JPG) [file pcbi.1012704.s004.jpg]

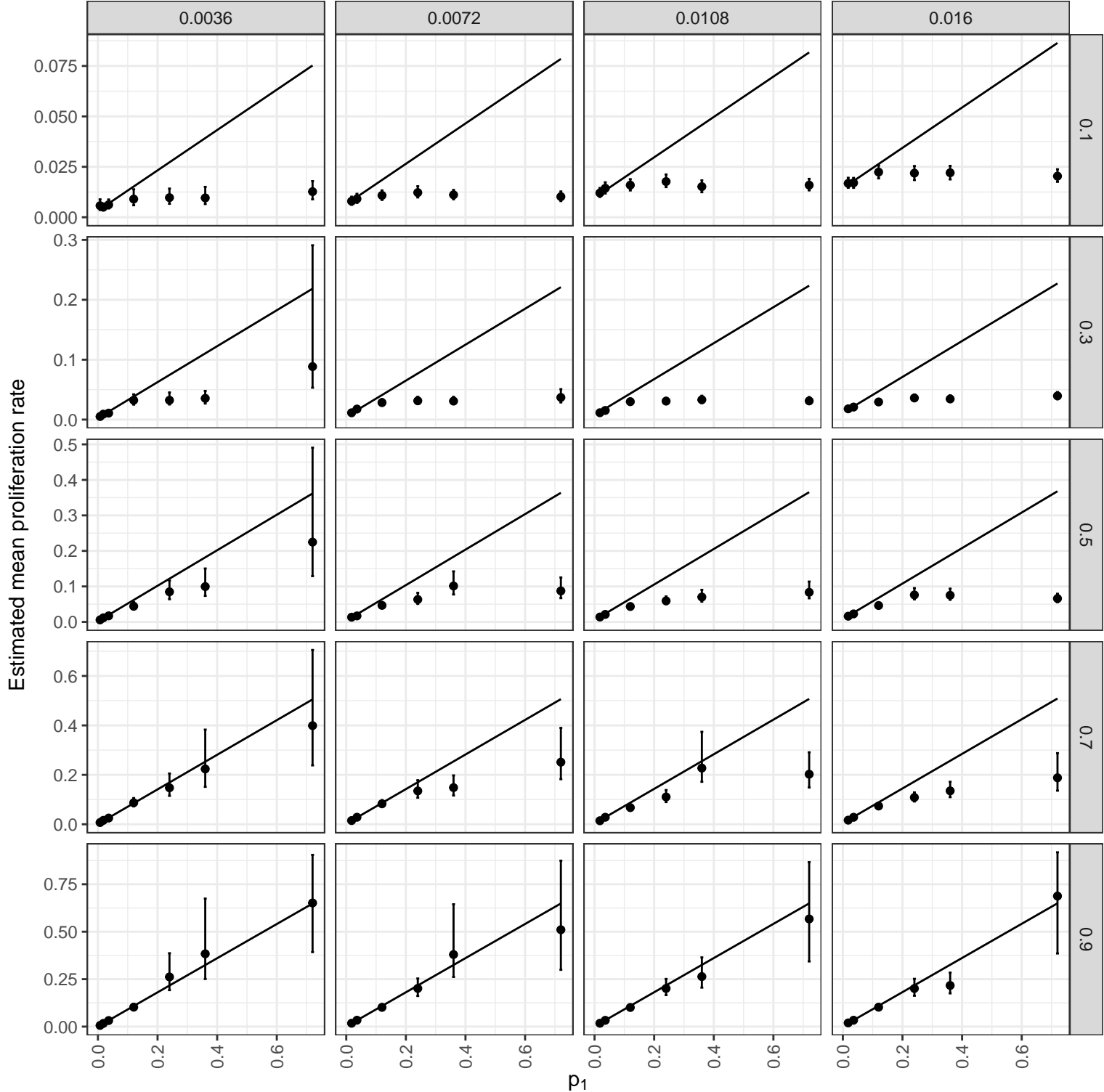

Supplement: S5 Fig — Estimated pE^ compared to (from left to right) the rates of proliferation, turnover and production by division of cells in the target compartment. Top: for the case when fitting the one compartment model; middle: for the case when fitting the precursor/target model bottom: for the case when fitting the precursor/target model with ratio. (PDF) [file pcbi.1012704.s005.pdf]

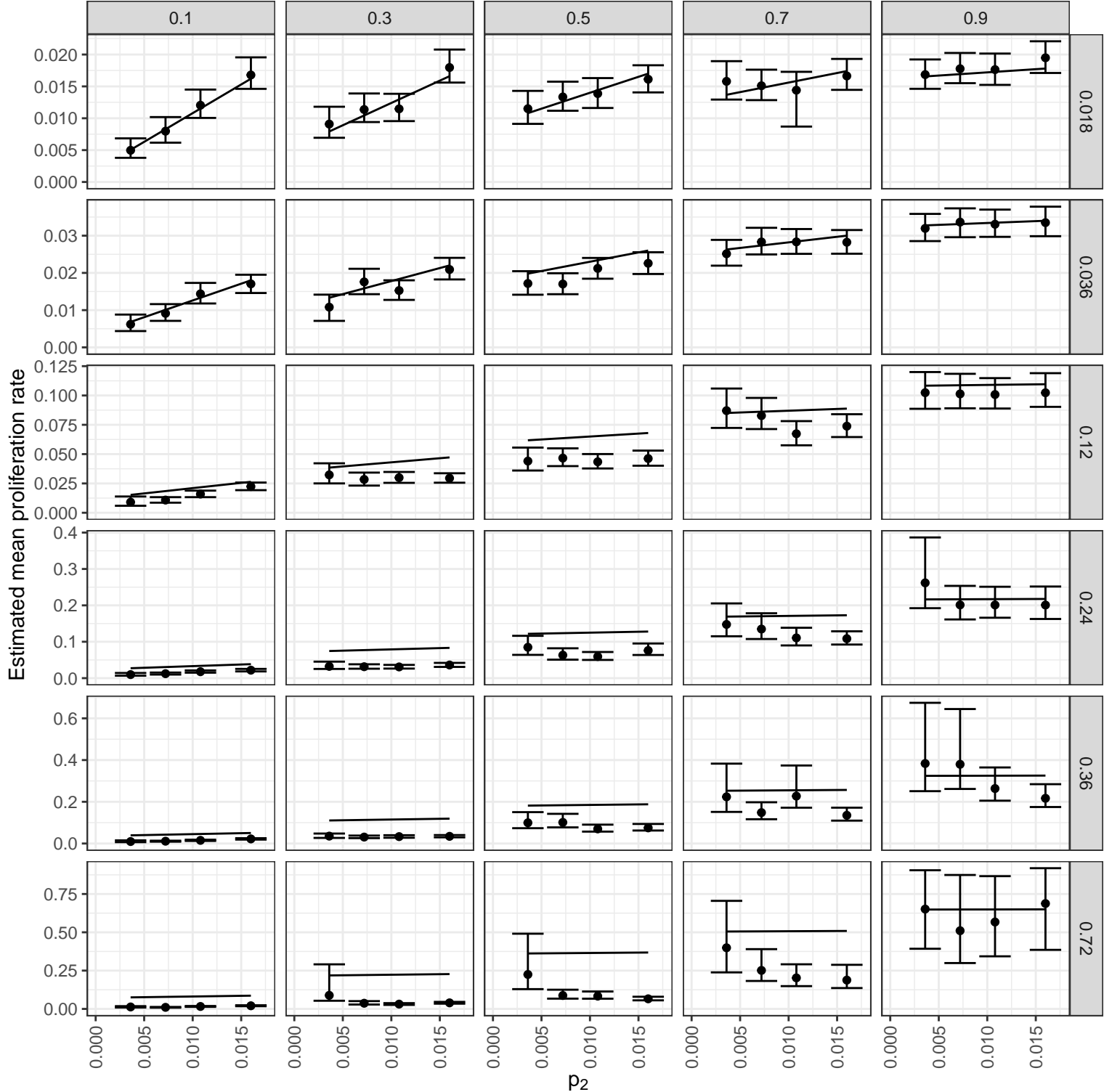

Supplement: S6 Fig — The value of p1 used to simulate the data is shown on the x-axis. Within each plot, the values of p2 and α1 used to simulate the data are held constant at the values on the top and right respectively. (PDF) [file pcbi.1012704.s006.pdf]

Estimated mean proliferation rate

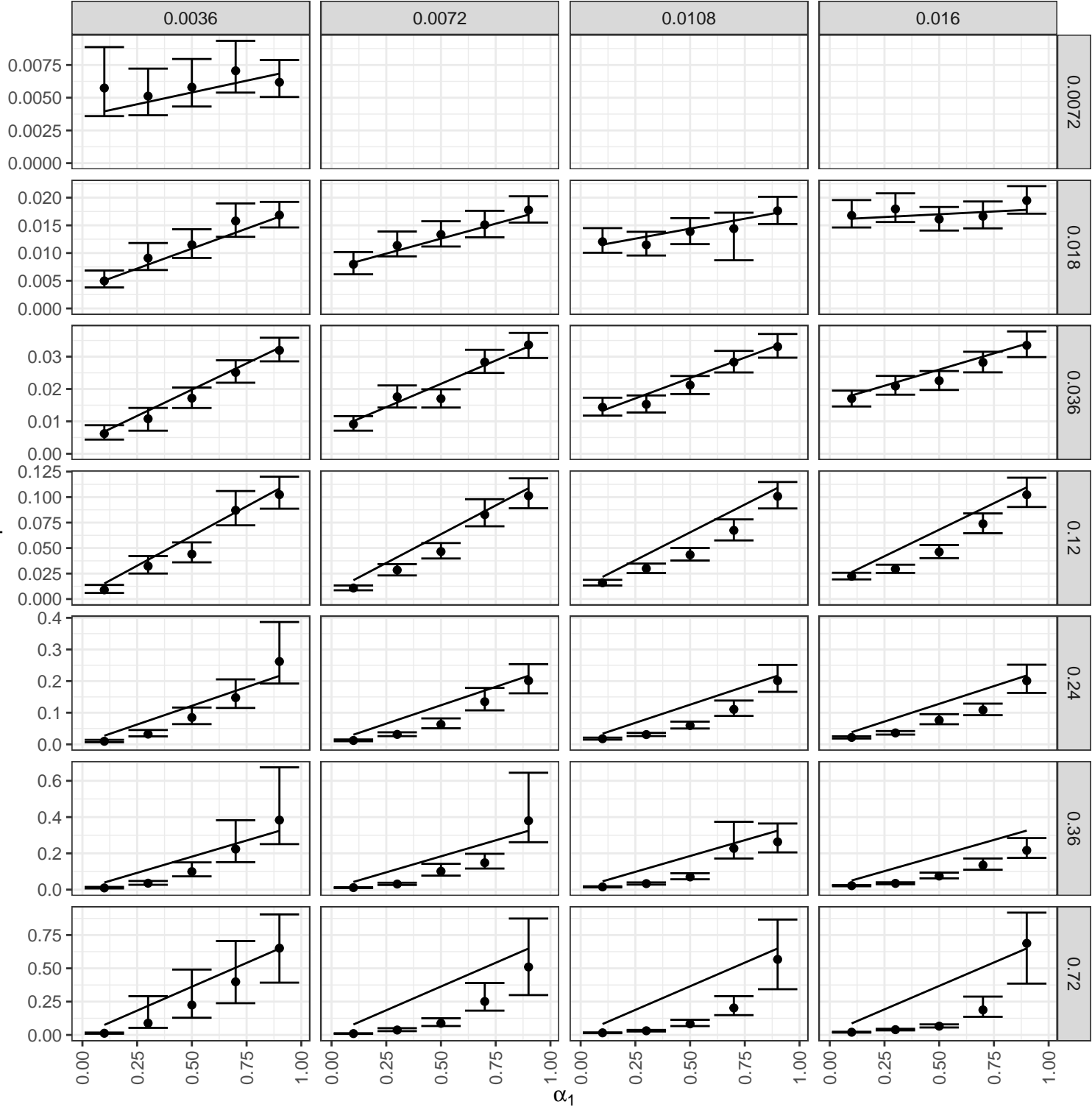

Supplement: S7 Fig — The value of α1 used to simulate the data is shown on the x-axis. Within each plot, the values of α1 and p1 used to simulate the data are held constant at the values on the top and right respectively. (PDF) [file pcbi.1012704.s007.pdf]

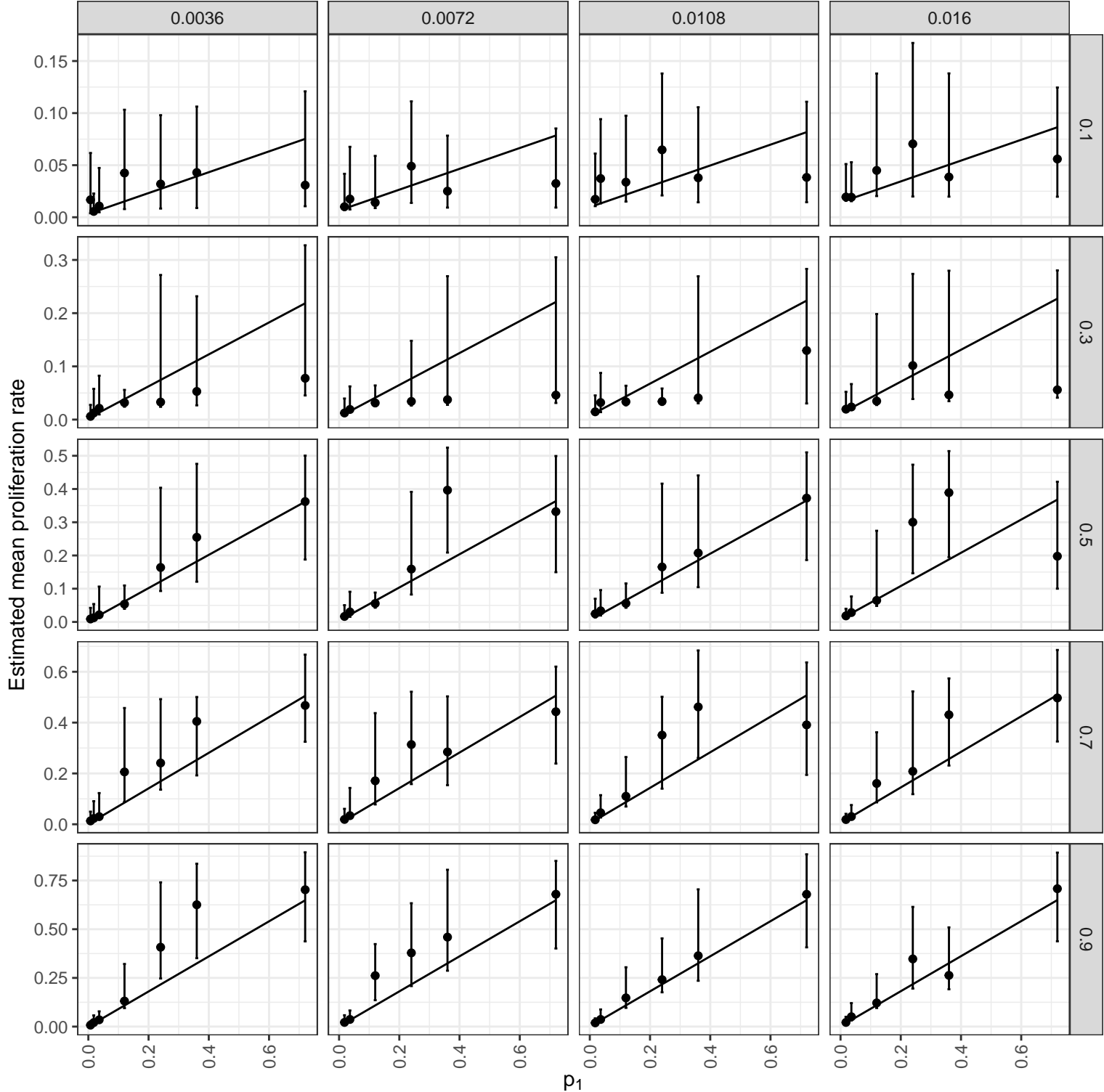

Supplement: S8 Fig — The value of α1 used to simulate the data is shown on the x-axis. Within each plot, the values of p2 and p1 used to simulate the data are held constant at the values on the top and right respectively. (PDF) [file pcbi.1012704.s008.pdf]

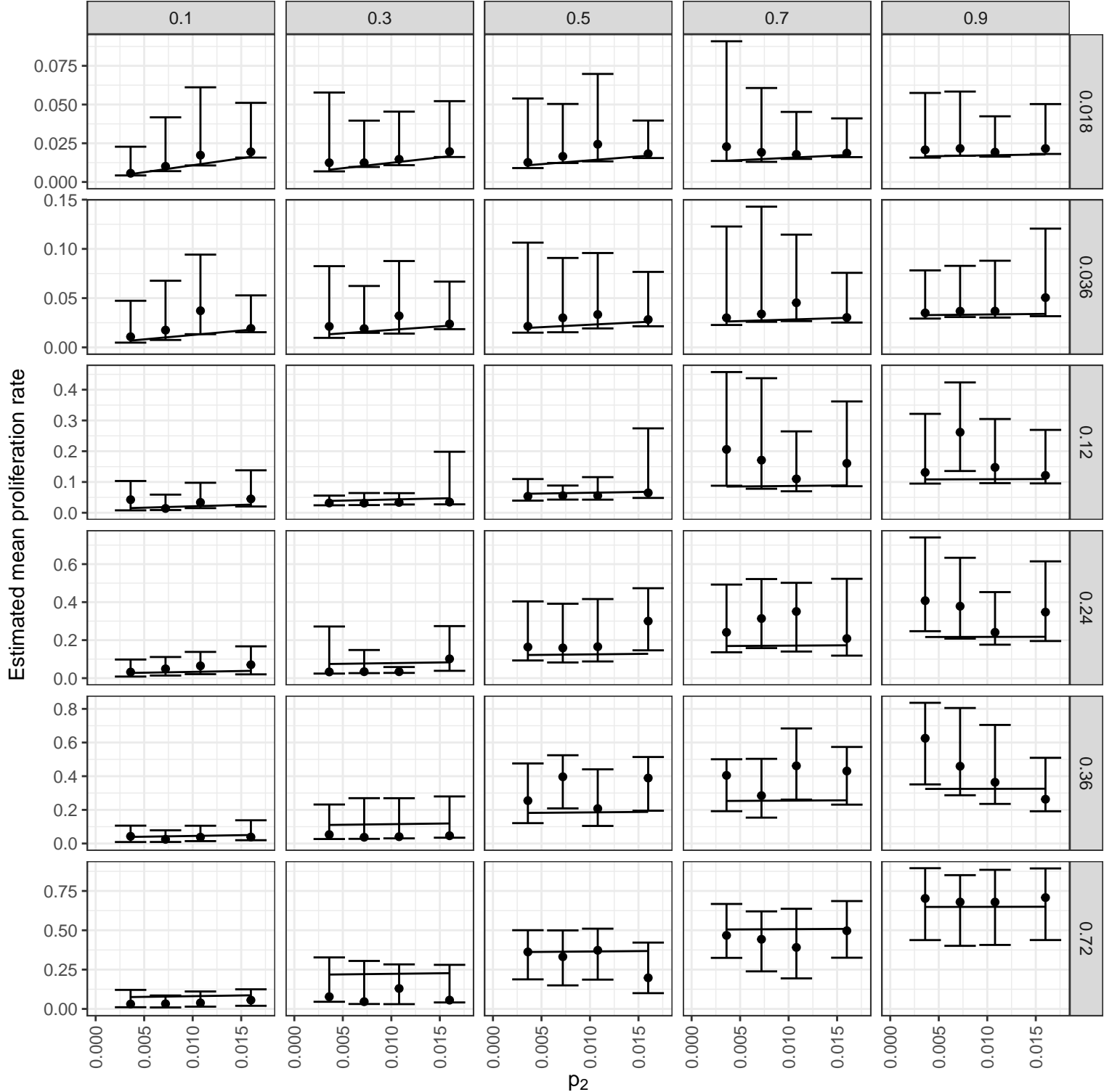

Supplement: S9 Fig — The value of p1 used to simulate the data is shown on the x-axis. Within each plot, the values of p2 and α1 used to simulate the data are held constant at the values on the top and right respectively. (PDF) [file pcbi.1012704.s009.pdf]

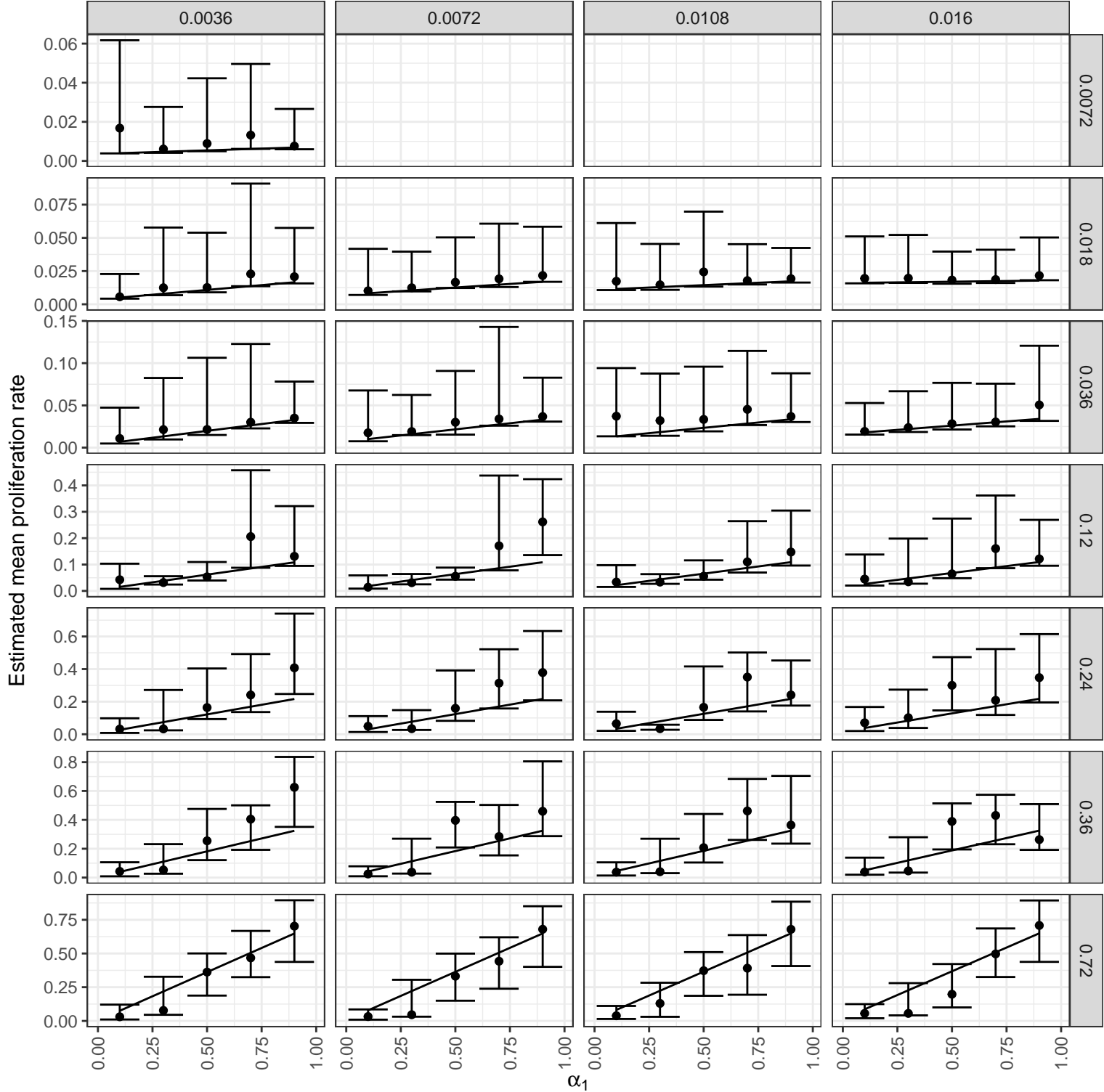

Supplement: S10 Fig — Median and 95% credible intervals for the mean proliferation rate when fitting the the two-compartment explicit model to data generated using the same model. The value of α1 used to simulate the data is shown on the x-axis. Within each plot, the values of α1 and p1 used to simulate the data are held constant at the values on the top and right respectively. (PDF) [file pcbi.1012704.s010.pdf]

$$p_1 = 0.72, p_2 = 0.016, \alpha_1 = 0.1$$

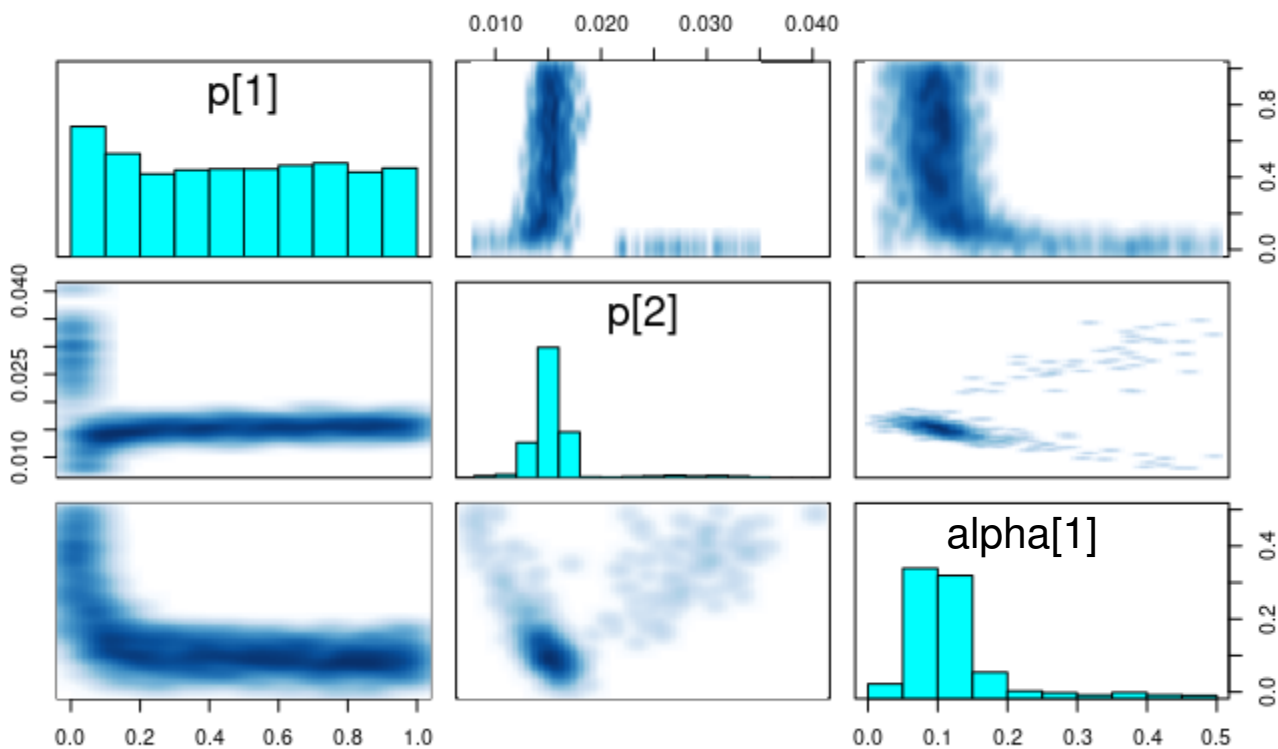

$$p_1 = 0.018, p_2 = 0.016, \alpha_1 = 0.1$$

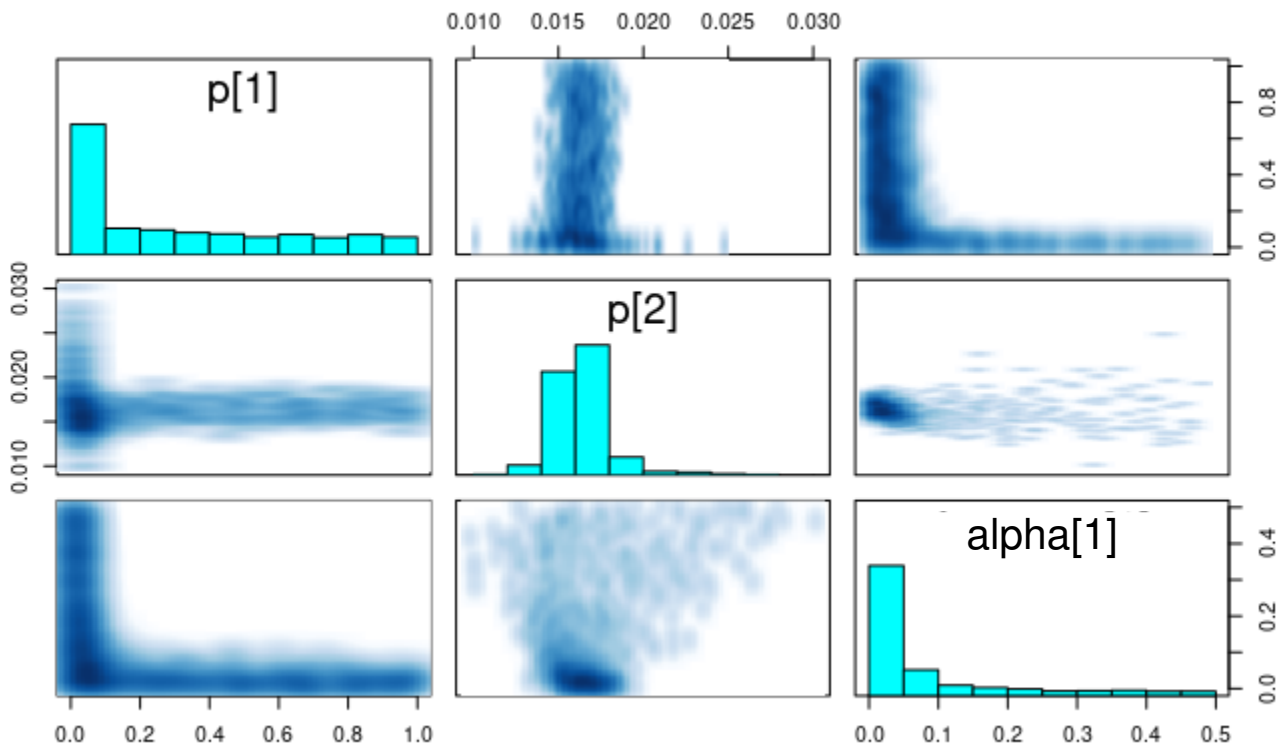

Supplement: S11 Fig — Median and 95% credible intervals for the mean proliferation rate when fitting the the two-compartment explicit model to data generated using the same model. The value of α1 used to simulate the data is shown on the x-axis. Within each plot, the values of p2 and p1 used to simulate the data are held constant at the values on the top and right respectively. (PDF) [file pcbi.1012704.s011.pdf]

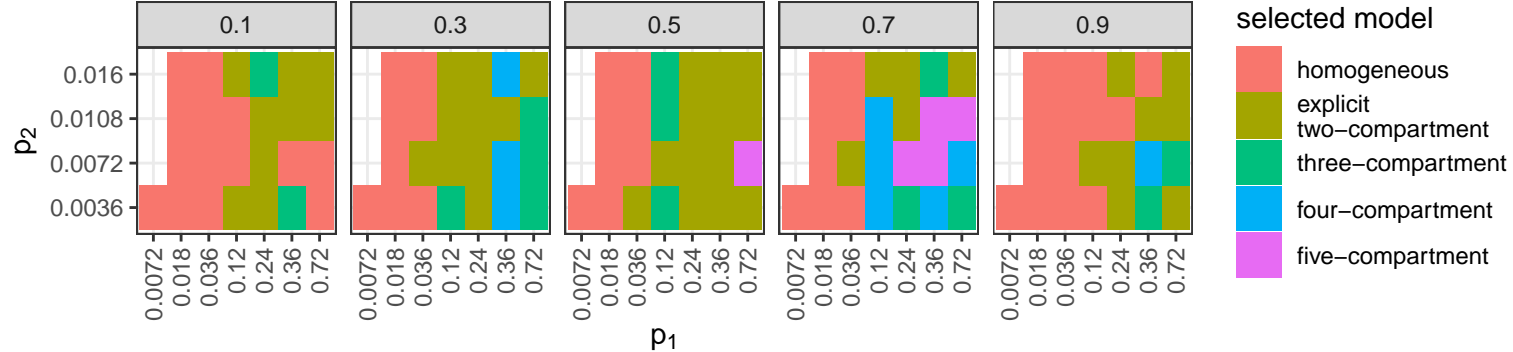

Supplement: S12 Fig — (PDF) [file pcbi.1012704.s012.pdf]

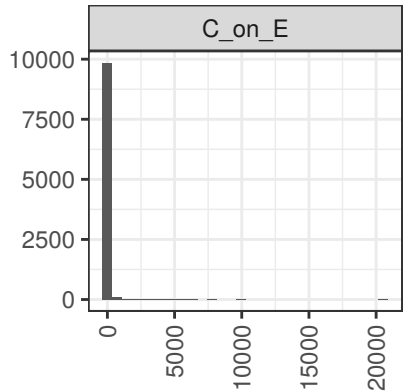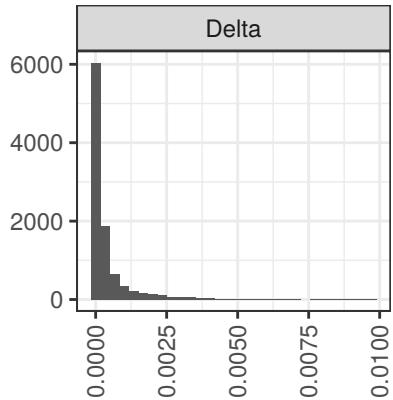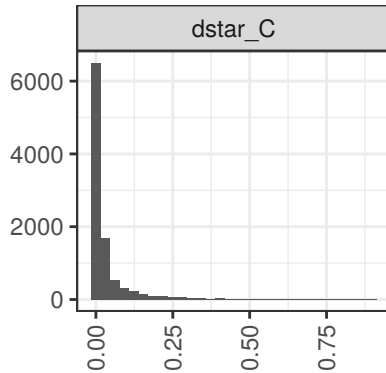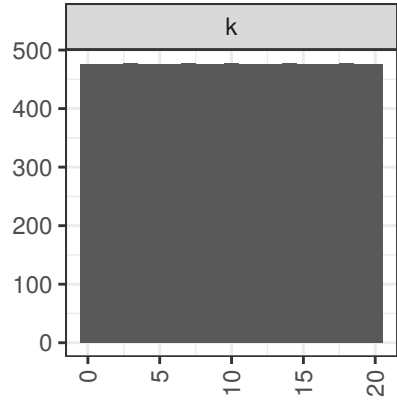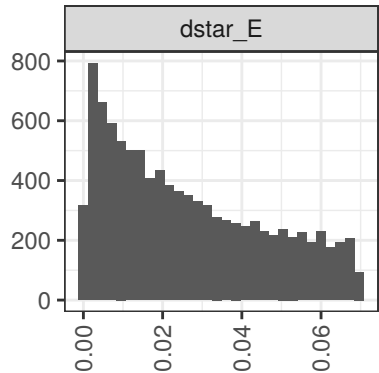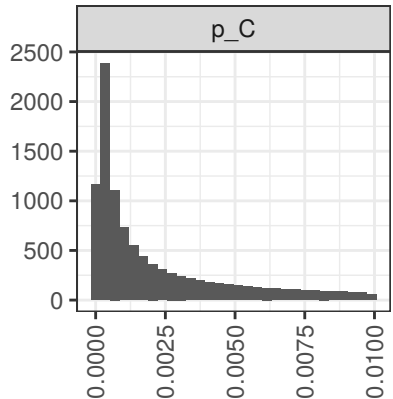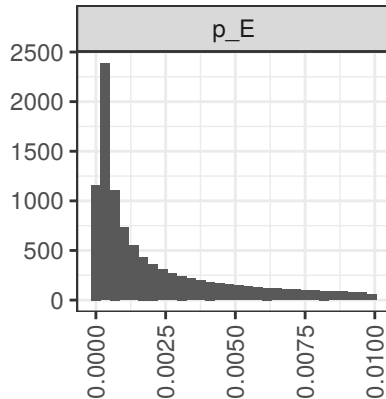

value

Supplement: S13 Fig — (PDF) [file pcbi.1012704.s013.pdf]
