# Supplementary material for: The impact of model assumptions in interpreting cell kinetic studies
Source: PLoS Comput Biol. 2025 Jun 3;21(6):e1012704. doi: 10.1371/journal.pcbi.1012704 (PMC12133179; doi:10.1371/journal.pcbi.1012704)
Supplement: S1 File — (PDF) [file pcbi.1012704.s014.pdf]

Supplementary Material

Derivation of approximation used in main text section

To recap the model equations in the main text, the fraction of label in the upstream and downstream compartments are

$$\frac{dF_C}{dt} = p_C b_w U(t) - (d_C^* + r)F_C, \tag{S1a}$$

$$\frac{dF_E}{dt} = (2^k - 1) b_w U(t) r \frac{\bar{C}}{\bar{E}} + r \frac{\bar{C}}{\bar{E}} F_C + p_E b_w U(t) - d_E^* F_E, \tag{S1b}$$

where

$$U(t) = \begin{cases} f[1 - \exp(-\delta t)] & \text{if } t \leq \tau \\ U(\tau)\exp(t - \tau) & \text{otherwise} \end{cases}, \tag{S2}$$

and the fitted model parameters obey

$$\frac{dF_E}{dt} = \hat{p}_E b_w U(t) - \hat{d}_E^* F_E. \tag{S3}$$

Comparing Eqs. S1 and S3, it is unclear how $\hat{p}$ and $\hat{d}$ relate to k, r, p_C, d_C, p_E, d_E, $\bar{C}/\bar{E}$. This is because F_C is a function of time.

We can analyse this system in two cases:

1. $2^k \gg 1$; and
2. $k = 0$.

1 $2^k \gg 1$

We establish an upper bound for $F_C(t)$:

$$F_C(t) = \frac{p_C b_w U(t) - \frac{dF_C}{dt}}{d_C^* + r} \tag{S4a}$$

$$\leq \frac{p_C b_w U(t)}{d_C^* + r} \tag{S4b}$$

$$\leq b_w U(t). \tag{S4c}$$

This result makes sense because the amount of label in cellular DNA cannot exceed the amount of label in body water (multiplied by the normalisation factor, which is the number of hydrogen atoms

in deoxyribose available to be replaced by deuterium).

It follows that

$$\frac{dF_E}{dt} \geq (2^k - 1)b_w U(t) r\frac{\bar{C}}{\bar{E}} + p_E b_w U(t) - d_E^* F_E \tag{S5a}$$
$$= \left[\left(2^k - 1\right) r\frac{\bar{C}}{\bar{E}} + p_E\right] b_w U(t) - d_E^* F_E, \tag{S5b}$$

and

$$\frac{dF_E}{dt} \leq (2^k - 1)b_w U(t) r\frac{\bar{C}}{\bar{E}} + b_w U(t) r\frac{\bar{C}}{\bar{E}} + p_E b_w U(t) - d_E^* F_E \tag{S6a}$$
$$= \left(2^k r\frac{\bar{C}}{\bar{E}} + p_E\right) b_w U(t) - d_E^* F_E, \tag{S6b}$$

As $2^k \gg 1$, the relative difference between $\left(2^k - 1\right) r\frac{\bar{C}}{\bar{E}} + p_E$ and $2^k r\frac{\bar{C}}{\bar{E}} + p_E$ is small. It is then reasonable to conclude, comparing to Eq. S3 that

$$\left(2^k - 1\right) r\frac{\bar{C}}{\bar{E}} + p_E \leq \hat{p} \leq 2^k r\frac{\bar{C}}{\bar{E}} + p_E, \tag{S7}$$

and

$$\hat{d^*} \approx d_E^*. \tag{S8}$$

2 $k = 0$

Production by division is defined to be $r(2^k - 1)C/E + p_E$ so in the case $k = 0$ this is equal to p_E, the proliferation rate of the target (downstream) population.

The full equation for F_E is given by the second equation of Eq. S1, which in the case $k = 0$ reduces to

$$\frac{dF_E}{dt} = p_E b_w U(t) - (d_E^* F_E - r\frac{\bar{C}}{\bar{E}} F_C) \tag{S9}$$

Given that well-defined numerical values of b_w and $U(t)$ are both entered into this equation then, if we fit the simplified equation, Eq S3, it can be seen that the estimated proliferation rate $\hat{p}$ will be a good approximation to p_E. It follows that the turnover rate $rC/E + p_E$ will be underestimated by $\hat{p}$. It can also be seen that the estimated disappearance rate $\hat{d^*}$ will approximate $d_E^* - r\frac{\bar{C}}{\bar{E}} F_C/F_E$, since the last term is always positive this will lead to an underestimate of d_E^*.

This is supported by our simulation-estimation study (Figure 3 D and H). In Supplementary Figure S1, we determine the error in $\hat{d^*}$ as a function of model parameters. We define the error to be the absolute value of the discrepancy between the true and estimated parameter values expressed as a proportion of the true parameter value

$$error = \frac{|estimated\ value - true\ value|}{true\ value} \tag{S10}$$

From the above, there is potential for d^* to be misestimated when $\frac{r\frac{\bar{C}}{\bar{E}}F_C(t)}{(2^k-1)r\frac{\bar{C}}{\bar{E}}b_wU(t)}$ is not close to 0. As $F_C(t)$ and $U(t)$ are time-dependent but $\frac{F_C(t)}{b_wU(t)} \leq 1 \quad \forall \quad t$, we conservatively use $\frac{r\frac{\bar{C}}{\bar{E}}}{(2^k-1)r\frac{\bar{C}}{\bar{E}}+p_E}$ instead (which is always below 1 for $1 \leq k \leq 20$). The top row of Supplementary Fig. **??** shows $\frac{r\frac{\bar{C}}{\bar{E}}}{(2^k-1)r\frac{\bar{C}}{\bar{E}}+p_E}$ versus the error in $\hat{d}^*$, for (A) $1 \leq k \leq 20$ and (B) $k = 0$. In both cases, when this ratio is below 1, the error in $\hat{d}^*$ is small (error < 5% for 99/100 simulations when $1 \leq k \leq 20$; error < 10% for 24/29 simulations when $k = 0$). Note that the denominator is the production rate by division. In practice, r and the true production rate by division are unknown. However, $r \leq p_C$ by model construction, and we may have some estimate of p_C (and $\frac{\bar{C}}{\bar{E}}$) if we know the identity of the upstream compartment. Also we can replace the true production rate by division by its estimate $\hat{p_E}$. The bottom row shows $\frac{p_C}{\hat{p_E}}\frac{\bar{C}}{\bar{E}}$ for (C) $1 \leq k \leq 20$ and (D) $k = 0$, with very similar results. We conclude that if we know p_C and $\frac{\bar{C}}{\bar{E}}$, then we can determine how likely there is to be error in d_E^*. It is possible that we do not have estimates of p_C and $\frac{\bar{C}}{\bar{E}}$ from the same system, but are only informed by prior estimates in the literature. The accuracy in p_C and $\frac{\bar{C}}{\bar{E}}$ needed to assess the error in d_E^* is the subject of future work.
