# Supplementary material for: The impact of model assumptions in interpreting cell kinetic studies
Source: PLoS Comput Biol. 2025 Jun 3;21(6):e1012704. doi: 10.1371/journal.pcbi.1012704 (PMC12133179; doi:10.1371/journal.pcbi.1012704)
Supplement: S3 File — (PDF) [file pcbi.1012704.s016.pdf]

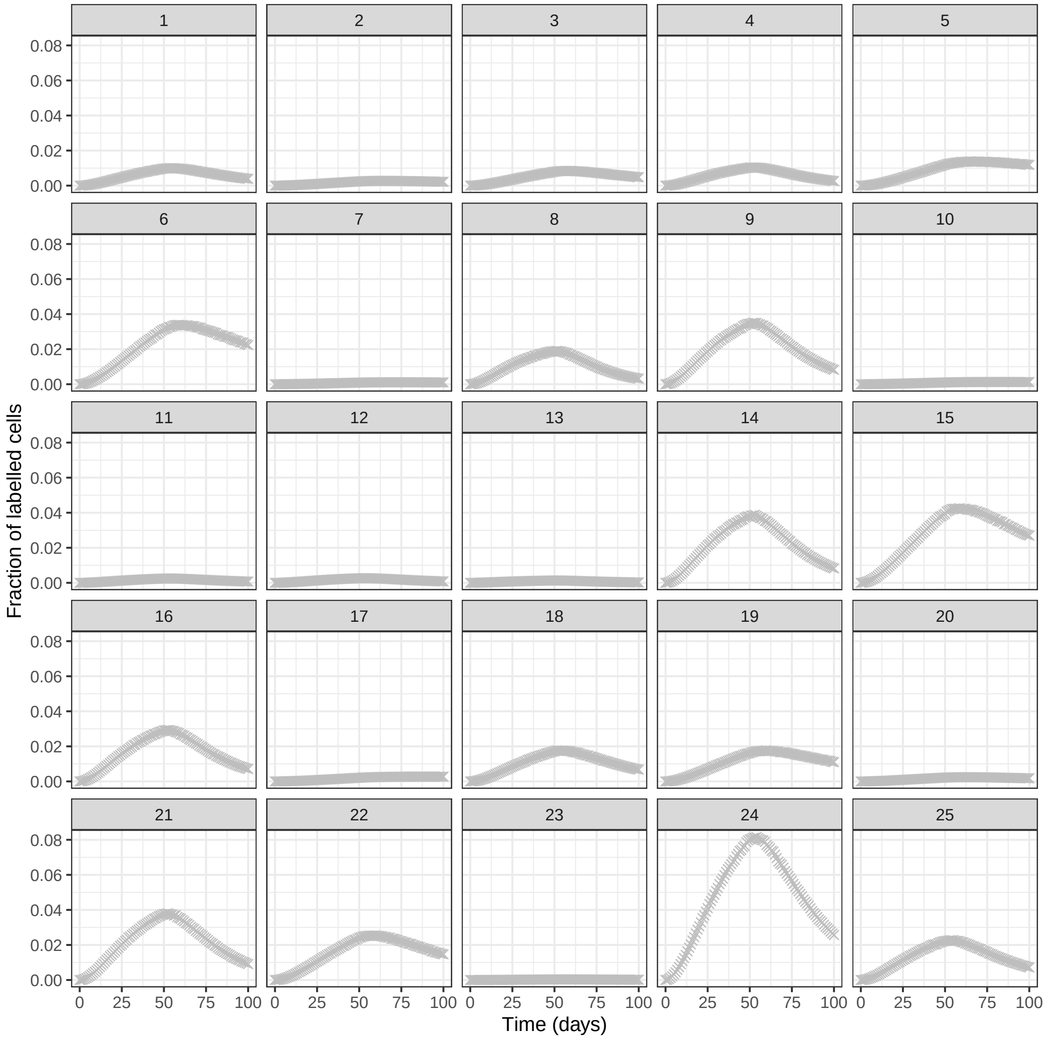

Figure A: Optimal data sets 1-25 (target compartment shown).

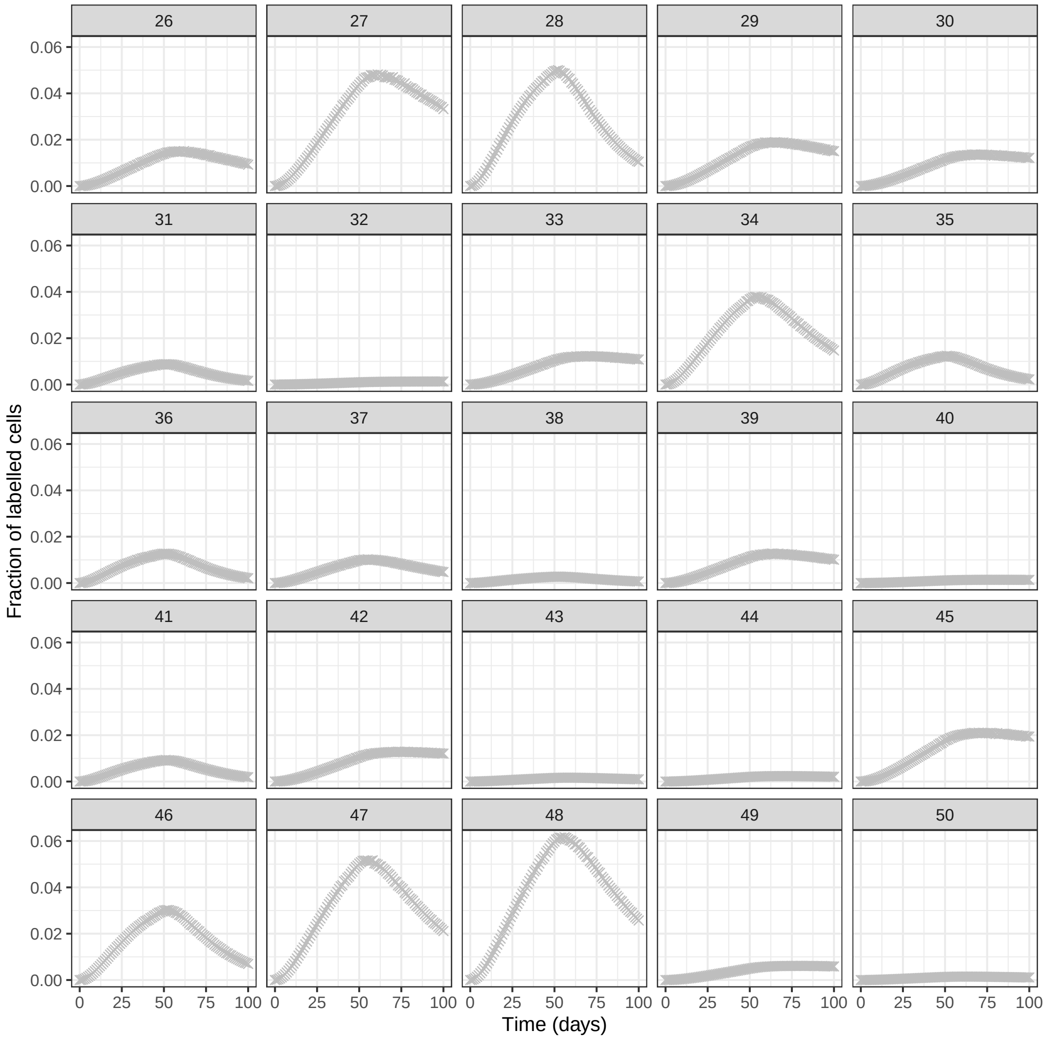

Figure B: Optimal data sets 26-50 (target compartment shown).

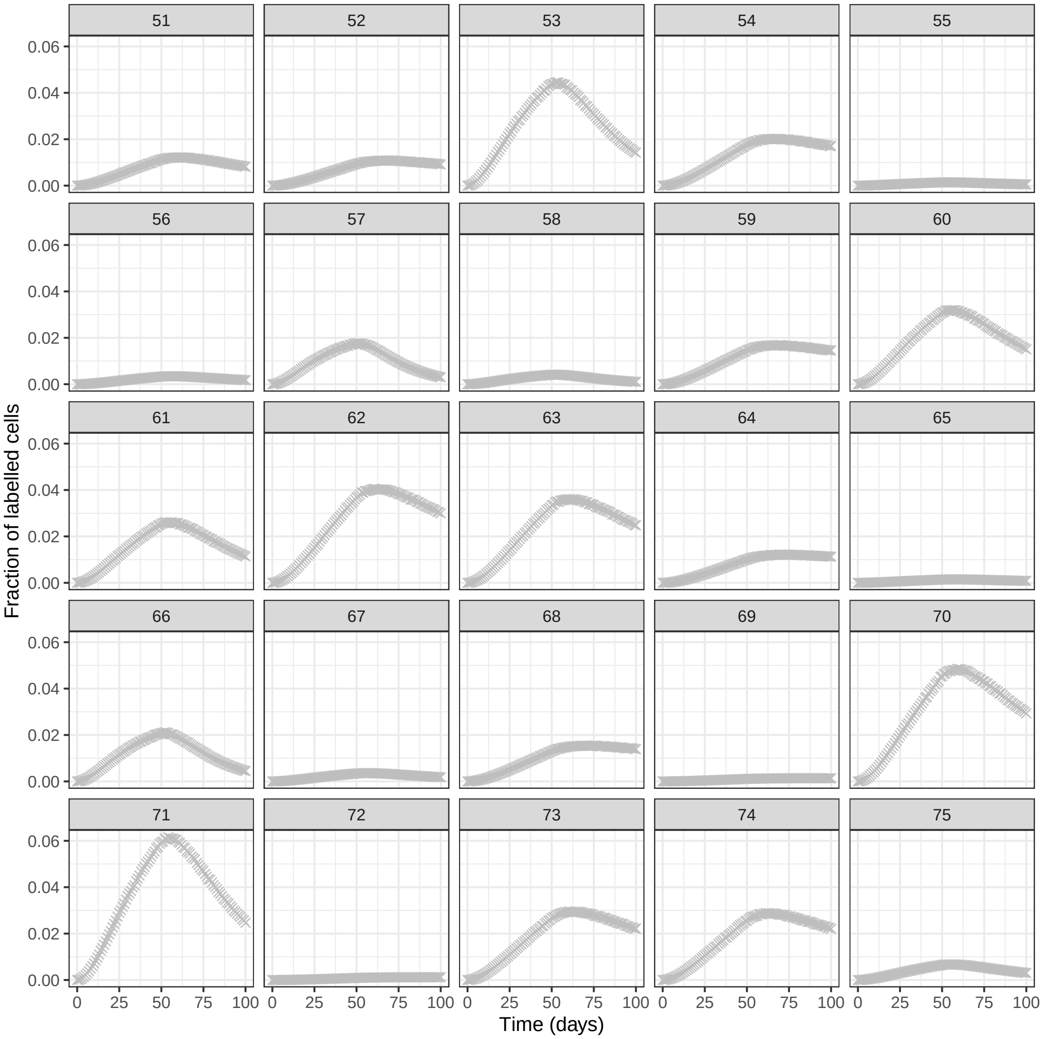

Figure C: Optimal data sets 51-75 (target compartment shown).

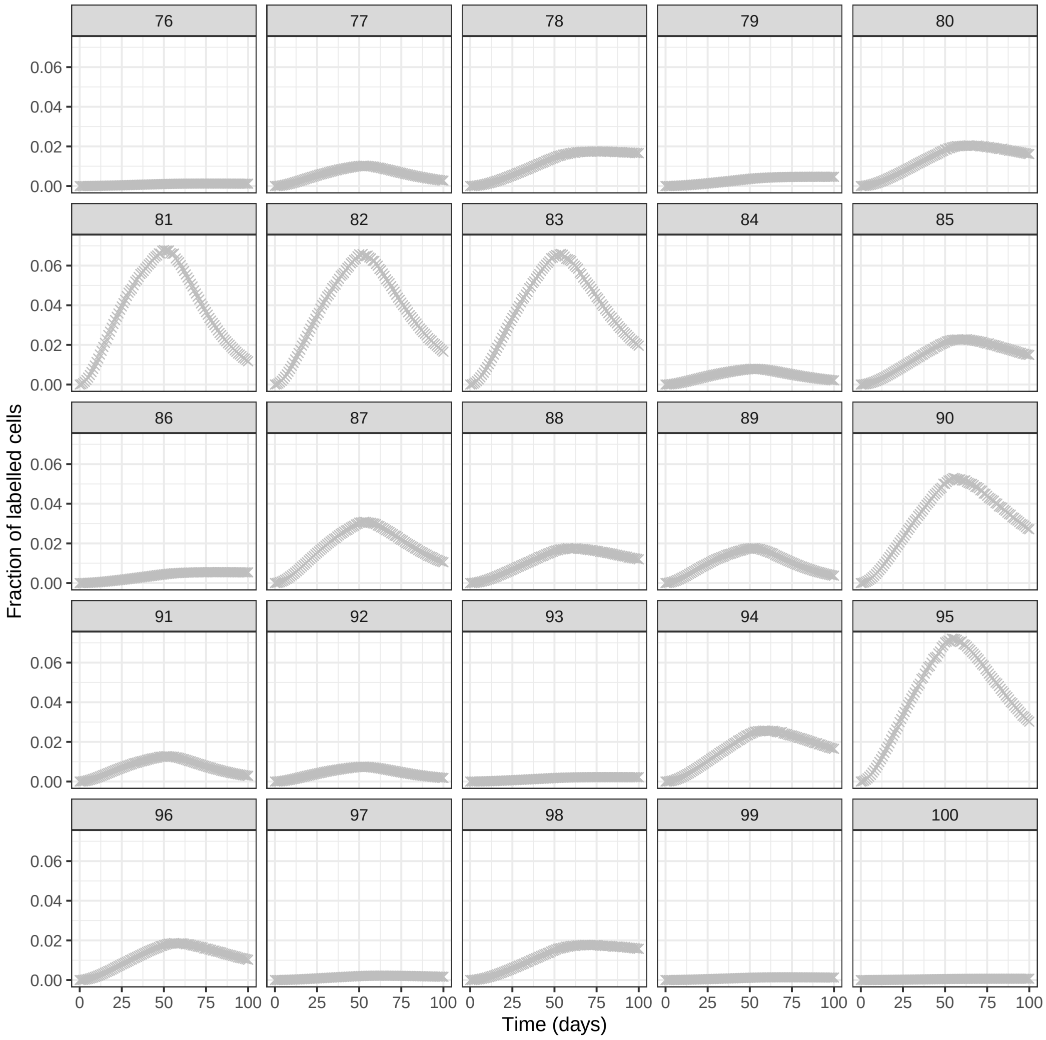

Figure D: Optimal data sets 76-100 (target compartment shown).

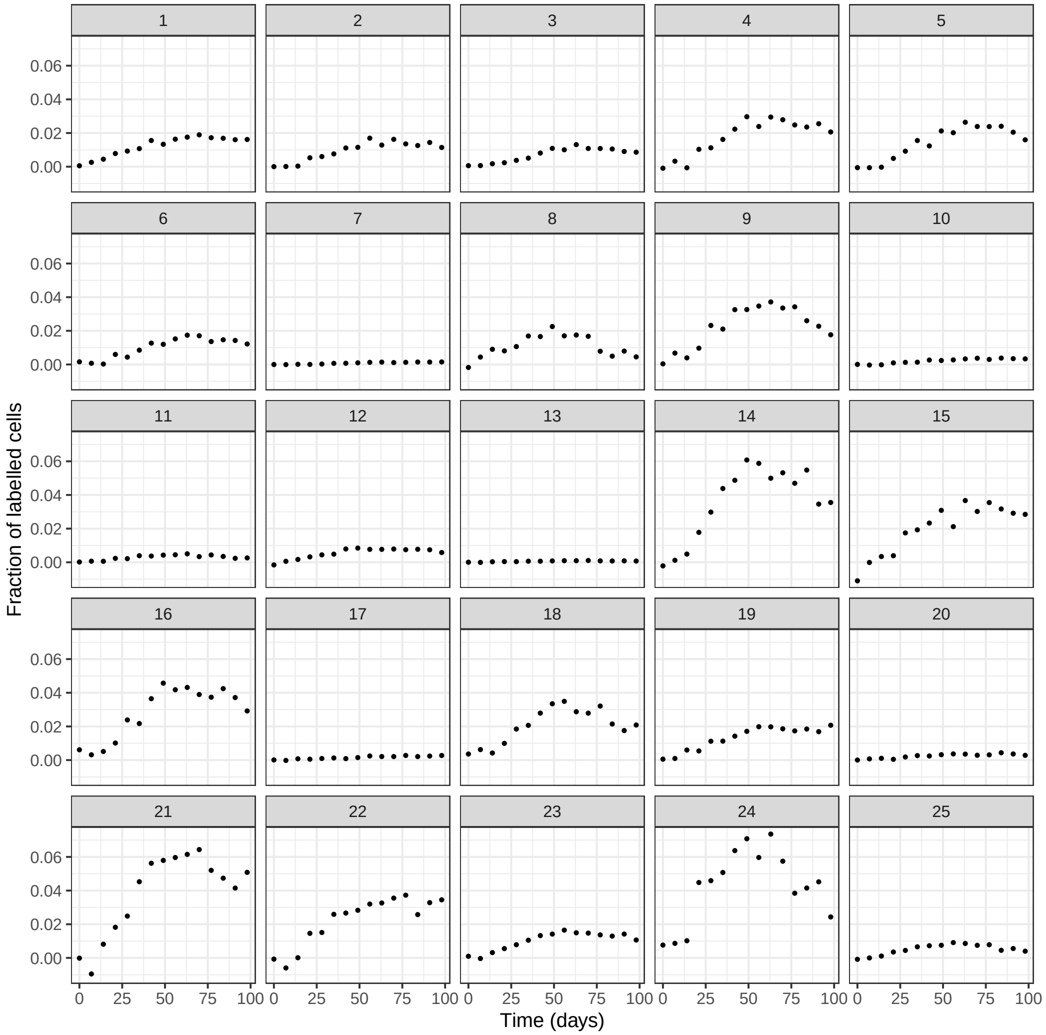

Figure E: Realistic data sets 1-25 (target compartment shown).

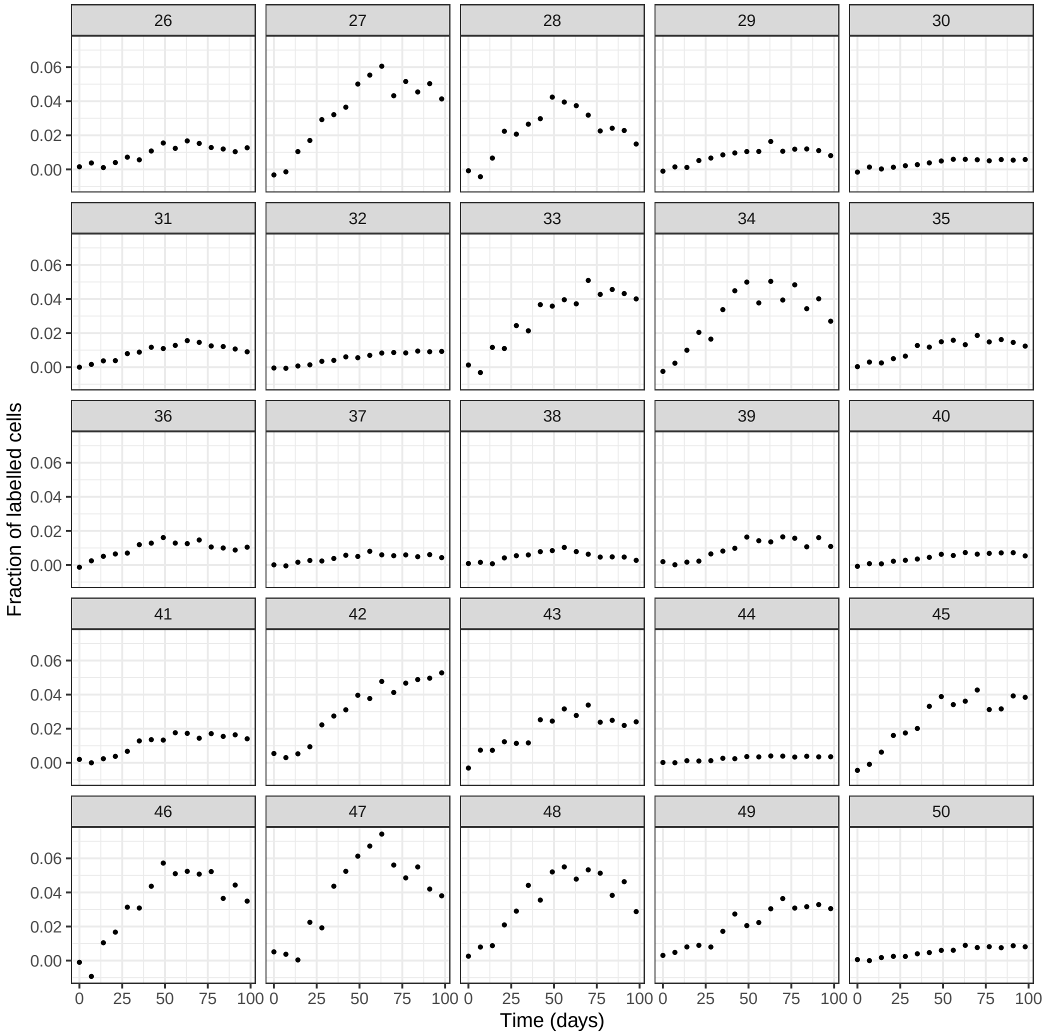

Figure F: Realistic data sets 26-50 (target compartment shown).

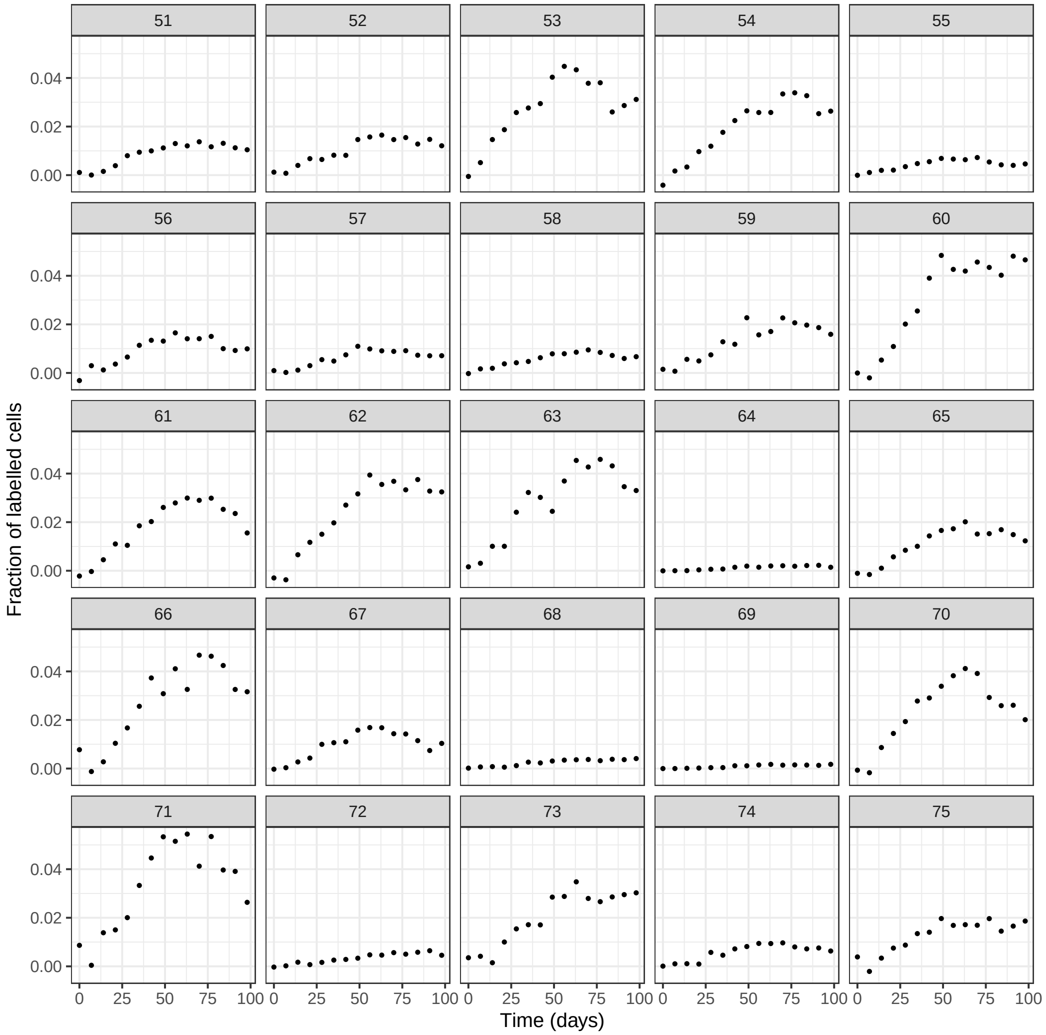

Figure G: Realistic data sets 51-75 (target compartment shown).

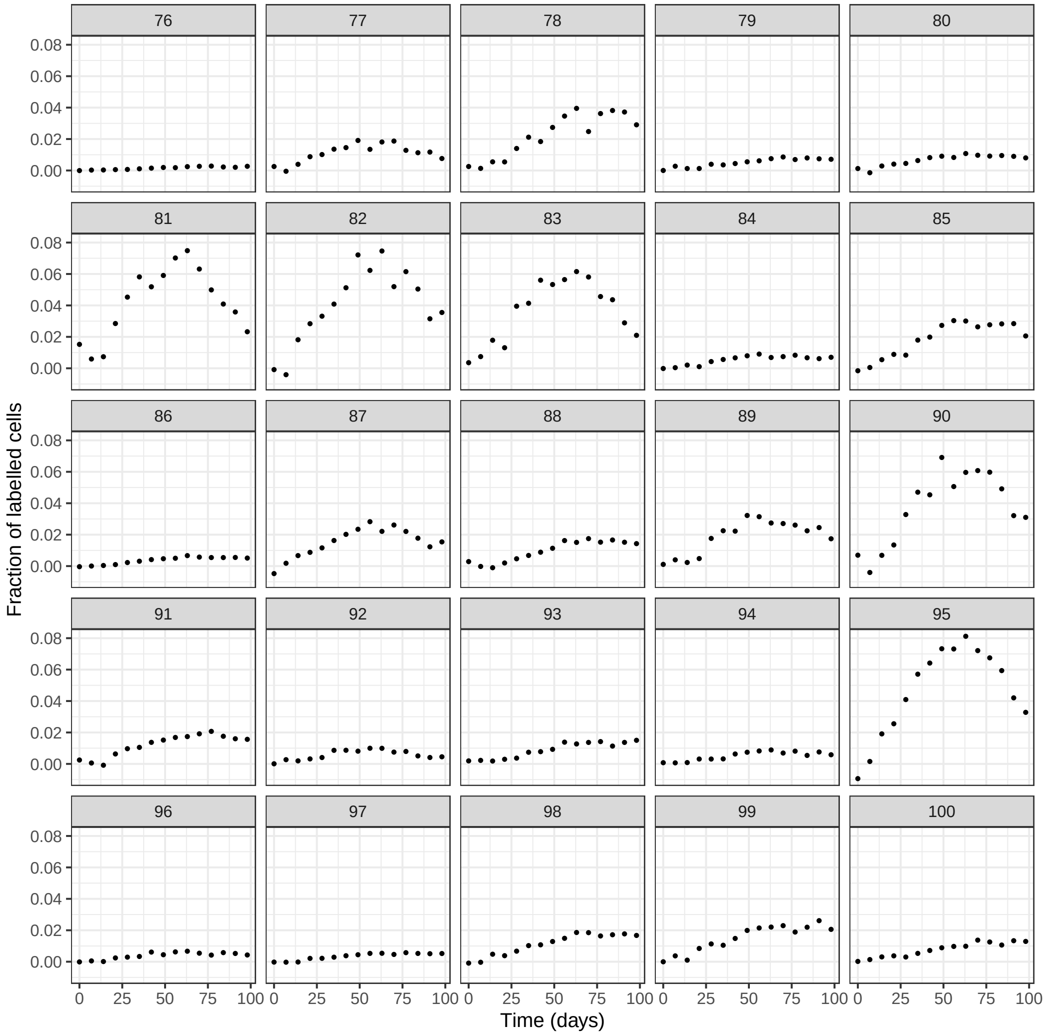

Figure H: Realistic data sets 76-100 (target compartment shown).
