# Supplementary material for: The impact of model assumptions in interpreting cell kinetic studies
Source: PLoS Comput Biol. 2025 Jun 3;21(6):e1012704. doi: 10.1371/journal.pcbi.1012704 (PMC12133179; doi:10.1371/journal.pcbi.1012704)
Supplement: Attachment — Submitted filename: response to reviewers.docx [file pcbi.1012704.s017.docx]

**RESPONSE TO REVIEWERS**

Thank you to all the reviewers for the helpful comments. We have extensively rewritten the manuscript to clarify it and to focus on a more intuitive understanding of the biases introduced by model misspecification. As we have made a number of changes to the manuscript the full tracked changes document is quite hard to follow so we have not uploaded it, instead we have uploaded a copy of the revised manuscript with major changes highlighted. If you would like the full tracked changes document then please do not hesitate to contact us.

**Reviewer #1:**

This is a thorough study exploring the caveats in the interpretation of deuterium labelling experiments.

It is obviously targeted at our fairly small community of labelling enthusiasts (!) but it still presupposes a lot of knowledge of the basics. It would be good to give a more detailed intro to the concepts - inheritance of label by DNA strands, etc.

We have now expanded the introduction to cover more of the basics –in particular the acquisition and loss of label compared to BrdU.

On the issue of where cells “go” between C and E. If E is fed by clonal expansion of C the transition is likely to be blurry - that is, cells may be classified as members of the target population while they are still dividing. If this is true it obviously becomes a bit of a mess - basically a KH model with a transitory fast-dividing state. This could be worth mentioning. This relates to the larger issue that in practice these processes you consider could be operating in tandem. Could you discuss how you might approach this issue?

We have added the following text to the manuscript:

In section 1 (the upstream/downstream compartment section), we avoid the issue of the transient identity of cells undergoing clonal expansion (i.e. do they contribute to label as phenotype C or phenotype E cells) by assuming clonal expansion occurs in the lymph node and that by the time we sample in the blood the cells can be classified as belonging to the downstream compartment, i.e. phenotype switching is complete. Thus, in this case the spatial heterogeneity in the system is to our advantage.

Regarding the processes operating in tandem, we have written:

More broadly, although we have discussed the processes of upstream and downstream compartments, kinetic heterogeneity and spatial heterogeneity separately, they may occur in tandem for a given situation. As there are many combinations of potential biases, we would first consider evidence for each of them separately. Given the complexity of the situation this evidence would need to be something other than the labelling data itself i.e. knowledge of the biological system.

Kinetic heterogeneity. The freedom here is assumed to be the number of independent compartments - but there are many possible relationships between kinetically distinct subsets - e.g. they might be related linearly, as a series of differentiated sub-states within the target population. Could you comment/speculate on whether this matters for your analysis?

This is a difficult question to address comprehensively as there are so many possible ways in which the subcompartments could be linked, and as we consider models with up to 6 subcompartments the space of models would be very large. Intuitively, we suspect that the results would be very similar –the basic problem of not being able to distinguish a large slowly turning subpopulation from a very small very fast subpopulation is unchanged. We have edited the text to make it clear that the version of heterogeneity we are considering is just one of many. We have also mentioned this point in the discussion.

Your Discussion summarises the results nicely. But I found the sections describing explorations of parameter spaces a little lengthy and hard to follow. I think the paper would read better if you condensed the numerical explorations and focused on more intuitive explanations of WHY you expect these biases to occur. I think it might also be informative to show correlations between parameters - so you can discuss identifiability as well as biases in parameter estimates.

We have condensed the section “Fitting a one-compartment model to the target data”, removing figure 2 and replacing it with an explanatory diagram of the biases due to assuming a closed population. We have also added a new table summarising our results on which parameters can be estimated in which scenarios (Table 1) as well as providing an intuitive explanation of these results (page 7 line 144 and page 8 line 171). Finally, we have edited this section heavily to try and improve clarity.

You ask about parameter identifiability and correlations. In most areas of this paper lack of identifiability is more to do with a mismatch between experiment design and the parameters of interest (e.g. in Section 1 due to the absence of data from the upstream compartment or in Section 2 due to labelling experiment design being fundamentally unable to identify small compartments with fast turnover) rather than a problem of model structure. The exception is the explicit kinetic heterogeneity model when strong correlations between parameters reduce identifiability. We have now added Supplementary Figure 11 showing these correlations.

it’s not 2H labelling but our study Lukas et al. Front Imm 2023 models the kinetics of division-linked labelling within the thymus and naive pool - our approach was to use an empirical description of the label freq kinetics within late-stage thymic precursors (your F_C (t)) to allow us to measure the turnover (loss rate and hence mean residence time) of naive T cells. For any given target population, we may have a few candidate precursor populations that can be treated in the same way, and fits to the target pop data with different precursors could be compared. We have also modelled dynamics of non-closed and kinetically heterogeneous target populations in several other studies (Gossel eLife 2017, Hogan eLife 2019, Bullock Plos Bio 2024, in press and currently on bioarxiv), which might be of interest.

Thank you for pointing us to Lukas et al (2023), we were not aware of this paper. Wouldn’t such an approach still be dependent upon having observations in the upstream compartment or knowledge of the parameters of this upstream population? We take the value of using an empirical rather than a mechanistic description if it results in fewer parameters but I don't think that this solves our fundamental problem of an absence of observations/knowledge of the kinetics of the upstream population. We have added a reference to Bullock et al. (2024) in lines 407: “Where possible, we should constrain parameter space using additional data, such as telomere length~\cite{borghans_telomere_2007, costadelamo_tscm_2018} or Ki-67 expression data~\cite{bullock_dynamics_2024}.”

Other minor points:

L82

2^k >>1 is the criterion for influx-dominated production. is it not more precise to say that it’s (2^k-1)b_w U(t) >> F_C ? In principle you could have a lot of clonal expansion (and hence label uptake) happening within the precursor but only a small fraction of it entering the target.

We have now rewritten this entire section being much more clear about the conditions under which the various kinetic parameters can be estimated.

L82-85

You’re dealing with the 1 compartment model here, so perhaps d^*_E should be just d_E?. Or clarify here in advance that you are always fitting the p d-star model

On line 108 we have added “In this study we will focus on the version of the model with implicit kinetic heterogeneity, as it is a more general version of the homogeneous model but retains parameter identifiability:”

L120

“The precursor cells turn over quickly” - now you use turn over in a different sense to your definition. Do you mean “the precursor cells proliferate extensively before entering the target compartment”?

The correct condition for the closed population model to yield accurate parameter estimates is that the precursor cells should have a high proliferation rate, and for the precursor population, the turnover rate is equal to the proliferation rate, so the definitions are consistent; though you make a valid point that this could be unclear so we have replaced turnover with proliferation

L132 “Unsurprisingly” - needs the detail on why you expect parameter identifiability issues before you say this

We have deleted “unsurprisingly”, as two sentences later we have “fit diagnostics indicate that parameters are not identifiable due to colinearity and the covariance matrix is singular” which discusses identifiability.

L338 What does “accurate assessment of prior data” mean? it would be great to include a more explicit recipe for how to proceed.

Whilst revising the manuscript we noticed that in the “Kinetic heterogeneity” section that you are referring to we had been analysing the absolute difference between estimated and true values, rather than the relative difference used in the rest of the manuscript. This introduced a bias towards large sampled parameter values in the analysis which we have now corrected. The new results actually show a much clearer and more decisive difference between the models and so we are able to give considerably more concrete recommendations for how to proceed.

L359 the number 2/98 needs a reference

We have now added a reference.

Lastly - I found the terminology a little confusing. This is not a huge issue as it’s mostly self- consistent, but I think it could be clearer. I think it’s more reasonable to describe self-renewal of existing memory cells as “division”, since most models describe this with single divisions. “Proliferation” is maybe better reserved for multiple divisions (clonal expansion)? For example, your phrase “rate of production by division” seems more closely aligned to self-renewal than influx from clonally expanded cells. And L87 - “then the proliferation rate is equal to the production rate by division” - this sounds true by definition.

Turnover is defined in other contexts as loss, so I advocate for a different terminology. Not required but food for thought:

Turnover = loss = disappearance (death plus differentiation)

Production = influx + self-renewal

And breaking it down further, influx = proliferation-linked influx + passive influx (or something similar)

At steady state, production = turnover

p_E is then the rate of self-renewal, not the proliferation rate

An alternative (and probably more useful) proposal is that you align the terminology with the companion paper from Swain et al.!

I guess one persons ”obvious” terminology is another person’s “totally opaque”! To me proliferation and division are synonymous, that one relates to multiple events and one to single events is entirely new to me! I think the best we can do is try and be very clear and internally consistent in our definitions. We have now added an additional figure (Figure 2) to show the relationships between definitions for clarity. I believe the terminology is also aligned between our paper and Swain et al.

**Reviewer #2:**

In this manuscript, the authors address the important question how necessary model assumptions when analysing data of isotope-labelling studies might influence the inferred kinetics of cellular turnover. In particular, they investigate the impact of three model assumptions, i.e. that the cell population is (1) from a single compartment, (2) kinetic homogeneous, and (3) spatially homogeneous. To this end they use simulated data and test the inference of the used parameters by subsequently fitting the different models to the data.

The study represents a valuable contribution to the interpretation of cellular turnover dynamics using isotope labelling data indicating potential scenarios of misinterpretation of parameters. The manuscript is well written and the argumentation can be easily followed. However, I would have some comments that the authors might want to consider.

\# Major points:

1.) In the analyses, the authors investigate to which extent the inference of cellular kinetics is impaired by inappropriate modelling assumptions for isotope labelling data. Most of the evaluations and comparisons are given in a qualitative way (i.e. "very similar", "much less", "a significant flow of labelled cells" etc) which partly makes it difficult to judge, under which conditions the inappropriate model assumptions clearly play a role., i.e., what exactly defines a significant flow. Given that they checked the robustness of the observations based on several parameter combinations (e.g. Figure 3) to show the generality of their observations, I was wondering if it would not be possible to make these statements a bit more quantitative, i.e., under which relationships of flux rates or loss rates you would expect which kind of deviation, which could help to assess potential correction factors.

This is a fair point. The problem though is that the size of the deviation varies considerably depending on the various parameter combinations and it is hard to summarise all the information accurately in words. We believe that the only way to accurately convey this is via figures. We have added some further figures –Figure 7, Supplementary Figures 1 &4 –to supplement the existing figures that show how error varies with the parameters (Figures 3, Supplementary Figures 5-10). In particular, in Supplementary Figure 1, we have summarised error for broad bands of the composite parameters to provide more of an overview. We have also tried to summarise some of the numerical findings in text lines 124 in the main text:

If we know the identity of the upstream compartment, we may have some information about $p_C$ (its proliferation rate) and $\frac{\bar{C}}{\bar{E}}$ (the ratio between the two population sizes) from the literature. As $p_C$ is an upper bound for $r$, $p_C \frac{\bar{C}}{\bar{E}}$ is an upper bound for the difference between the rates of turnover and production by division. However, without knowledge of $k$, it is difficult to find a bound for the difference between the rates or proliferation and production by division.

2.) Equation 1 and line 49: I would strongly recommend to replace the symbol $\Delta$ (capital delta) for the differentiation rate as this will be easily misread as the Laplace-Operator within differential equations.

That is a good point. We have now replaced Δ with r throughout.

3.) Within the spatial model, the authors assume a recirculation of cells between lymphoid tissue and peripheral blood finding mostly an overlay between the dynamics of labelled cells within the two compartments (e.g. Figure 12), i.e. indicating a reasonable interpretation of measurements within this context. However, the model neglects any loss of cells from the blood (e.g. migration of cells to peripheral tissue), which would remove cells from the circulation and could also happen at a different rate than the disappearance of cells from the lymphoid tissue. I think this could be a relevant factor when considering physiological conditions and might be worth to discuss or investigate.

Long-term loss of cells from the circulation to peripheral tissues would be captured in the quantified disappearance rate (together with long term loss to lymphoid tissue, death and phenotype switch).

\# Minor points:

- Within the figures the axis labelling and axis annotations are partly very difficult to read (e.g. Figure 4)

We have now been through the manuscript checking the figures for clarity in particular we have corrected Figure 4 and Supplementary Figure 2.

- Figure 4: Were the statistical tests used for comparison corrected for multiple comparisons?

No p values are not corrected for multiple comparisons, we have now clarified this in the manuscript and added the number of comparisons made.

**Reviewer #3:**

In this manuscript, authors assess the impact of three model assumptions for fitting stable isotope labelling data. They use simulated data to investigate the impact of model assumptions on parameters estimations and the accuracy of these estimations. I found the paper easy to follow, however I think it could be streamlined by reducing some sections and better highlighting the new results. For me it's unclear what are the biological implications of this work and the impact in isotope labelling experiments. It does not seem that the authors come up with specific methological recommendations based on their results (except "balancing accuracy with precision", which is pretty vague).

We have now tried to make more explicit recommendations for the “Upstream and Downstream Compartments” and “Kinetic Heterogeneity” sections. In particular, whilst revising the manuscript we noticed that in the “Kinetic heterogeneity” section we had reported the absolute difference between estimated and true values, rather than the relative difference used in the rest of the manuscript. This introduced a bias towards large sampled parameter values in the analysis which we have now corrected. The new results show a clearer and more decisive difference between the models and so we are able to give more concrete recommendations for how to proceed. We have added the following text to the two sections:

“Upstream and Downstream Compartments”:

In summary, we recommend that where it is suspected that there may be an upstream compartment but this compartment has not been sampled then the one compartment implicit kinetic heterogeneity model \ref{eq:one_compartment_model} is fitted and the estimated parameter $\hat{p}$ is interpreted as the production rate by division. This interpretation will always be correct independent of the dynamics or relative size of the upstream compartment. If $\hat{p}$ is equated with proliferation and/or turnover then the necessary assumptions should be spelt out (that there are no labelled cells entering from an upstream source and that there are no unlabelled cells entering from an upstream source respectively). Ideally, further information should be obtained about the size and dynamics of the upstream population. In the case where there is considerable label entering from the upstream compartment and we wish to quantify how much of the production by division is attributable to the proliferation of the target population then an improved estimate of the proliferation can be made by fitting the precursor/target model though this may suffer from large errors.

“Kinetic Heterogeneity” section

We thus recommend choosing a model based on prior data rather than through model selection. If prior data is limited or uninformative then the implicit model should be used, but we should be aware that this model assumes a small degree of heterogeneity; greater heterogeneity may be present but undetectable in the data. On the other hand, if an informative prior can be generated, an explicit $n$-compartment model may be better. Where possible, we should constrain parameter space using additional data, such as telomere length or Ki-67 expression data.

Please find some detailed comments below:

- overall comment: could you make a table that summarises the questions asked / results obtained? In the intro it seems that you’ll only address 3 points but some are divided in further sub-questions and it would be nice to get an overview of this

We introduced such a table for the first section (Figure 5) but felt the other sections were simpler and so did not really require such an overview. Please let us know if you disagree and we can include an additional table.

- is paragraph lines 76-89 necessary? I find it very vague and approximate. Could it be reduced and used as quick introduction for the following paragraph?

We have rewritten these lines to be more precise, focusing on explaining the source of bias in parameter inference. The new text reads:

In Eq~\ref{eq:one_compartment_model}, proliferation, production by division and turnover are described by a single rate $p$. This will evidently cause problems when fitting to the target data, as Fig~\ref{fig:source_term_summary} shows that these terms are not equal for the target compartment. In \nameref{S1_File}, we show that the estimated $p$ (which we will refer to as $\hat{p}$) most closely approximates the production rate by division, which for $2^k \gg 1$ is approximately equal to the turnover rate, and for $k = 0$ is approximately equal to the proliferation rate. Thus for $2^k \gg 1$, the proliferation rate is underestimated by $\hat{p}$, and for $k = 0$, the turnover rate is overestimated by $\hat{p}$ as summarised in Fig.~\ref{fig:source_term_summary}. As the error occurs due to the conflation of the proliferation rate, production rate by division and turnover rates, its magnitude is equal to that of the true discrepancy between these rates in the target compartment, which is not possible to assess with information from the target compartment only.

- section starting line 90: could you comment (maybe in the discussion) on the error margins here and how much of an impact it makes in isotope labelling studies? (e.g., Fig 2 if the true value is 0.007 and the estimate around 0.002, what does it mean in terms of interpretation of results in a given labelling study, and what are the implications of such error?)

The implication of the error very much depends on the question being addressed and the size of the error. This would need to be considered on a case by case basis. It is important to note that the “error” in the upstream/downstream section is not so much an error as misinterpretation of a parameter. Provided $\hat{p}$ is interpreted as production by division then there is no error.

- Lines 211-224 is more “introduction” and not really results. I would reduce to focus more on the new results presented by this paper. It's also unclear what results are new in paragraph lines 211-240.

We have deleted lines 211-224 and lines 238-240 to accentuate the main result: “This work (Figure 6) illustrates a fundamental problem with estimating the mean proliferation rate using stable isotope labelling: that it is difficult to rule out the existence of a fast, small compartment which, while not changing the overall proportion of labelled cells much, does drive up the mean proliferation rate.”

- Line 218: you mention "underestimation of mean proliferation rate". Fig D shows over-estimation of this rate, does that mean that your results are not in line with Westera et al?

Both Westera et al. and our Fig. 7 show underestimation of the mean proliferation rate by the homogeneous and implicit kinetic heterogeneity models (models “1” and “implicit” on the x-axis in Fig. 7). Our results are in complete agreement with Westera et al, our point is that they use just one parameter combination and an informative prior in reaching their conclusions; we get identical results to them for this parameter combination and prior.

- Figure 6: could you try to use same scales for the y-axis? I find the comparison A-D, B-E, C-F difficult to make visually

We have now deleted this figure.

- Lines 255-256 "This indicates that the estimates of mean proliferation rate obtained using the explicit heterogeneity models are heavily dependent on prior assumptions": I think to state this you would need to check the results for more than just 2 priors

We have now repeated the analysis for more parameter sets with additional uniform priors. We now show (Figure 8C and 8D) errors for priors spanning [0,1], [0, 2], [0, 5] and [0,10]. These additional runs yield very similar results. We have restricted our analysis to uniform priors as these are the most commonly used.

- Lines 270-271, 275, when you mention the "lower bound" and "upper bound". I am not convinced by these claims. How helpful is a lower/upper bound if we don't know how accurate this bound is? For example if I read Fig 8 properly, the proliferation rate estimation can be around 0.5 in some cases, while the parameter is 0.0036. I'm not sure this upper bound value is helpful in any way.

This section has been deleted. Though the earlier section (upstream/downstream compartments) we do make statements about bounds on parameters. We take your point that large bounds may not be very useful nevertheless we are trying to make concrete recommendations about what can and what cant be learnt from labelling studies.

- Lines 278-289: is this more for the discussion?

This section has been deleted.

- line 151 you mention 100 simulated datasets (maybe refer to the methods here, as there is no mention of any simulated datasets before in the results section), but line 301 you refer to 125 simulated datasets. I don't find any mention of 125 simulations in the methods. Could you please clarify?

In the section *Upstream and downstream compartments,* we simulated 100 datasets from the upstream/target model; we have now added “The top row shows results for $1 \leq k \leq 20$ for 100 simulated data sets (Methods)” on lines 130 to reference this earlier. In the section *Kinetic heterogeneity*, we separately simulated 125 datasets from the explicit kinetic heterogeneity model; we have now clarified this in the methods.

- minor: some figures have titles, some not. Please harmonise

Apologies for this, all figures now have titles.
