# Supplementary material for: The impact of model assumptions in interpreting cell kinetic studies
Source: PLoS Comput Biol. 2025 Jun 3;21(6):e1012704. doi: 10.1371/journal.pcbi.1012704 (PMC12133179; doi:10.1371/journal.pcbi.1012704)
Supplement: Attachment — Submitted filename: source_term_response_reviewers_comments_round_2.docx [file pcbi.1012704.s018.docx]

Thank you to all the reviewers for the helpful comments.

Reviewer #1: Thanks to the authors for the careful responses, and the extensive and clarifying revisions.

Reviewer #2: The authors have performed additional analyses to address the comments brought forward by the reviewers, substantially revising the manuscript. Although there seems to be an issue with the Figure numbering in response to my previous comments, the authors have addressed them appropriately. I see the difficulty of providing general relationships and correction factors to avoid misinterpretation of parameter estimates, which slightly limits the applicability of the study, but nevertheless raises the awareness of the misinterpretation problem and strategies to overcome them. I would only have one minor comment which concerns the statistical analyses, as e.g. performed in Figure 6:

If I understand correctly, the statistics and comparisons (as e.g. Figure 6A) are performed on 100 simulated data sets of the two compartment model using "a wide range of parameters" (line 131). Given that Eq.2 contains several free parameters, I think it could be relevant to check that the 100 parameter combinations truly cover a wide range for each of the parameters used, i.e. by showing the distribution. How many parameters were actually varied? It is not directly clear if 100 parameter combinations are sufficient to address a large range of parameter combinations. In addition, do these parameter combinations generally lead to "informative dynamics", e.g. not immediate loss of all cells, which might affect the ability to infer meaningful dynamics? This is only a minor point which could affect the comparisons, but I apologise if this has already been addressed and I missed this within the text.

All parameters were varied using Latin hypercube sampling (lines 548-549). We have now added S2 File and S3 File with the parameter sets and plots for the simulated data respectively, showing that the simulated data covers a range of possible dynamics.
